# Pyrogallol Contributes to the Anti-Allergic and Anti-Inflammatory Activities of Rosebud Extracts of Newly Crossbred Roses

**DOI:** 10.3390/cimb48050448

**Published:** 2026-04-25

**Authors:** In-Jeong Kim, Khan-Erdene Tsolmon, Zolzaya Bavuu, Seung Tae Kim, Solar Sora Kim, Dongsun Park, Yeon Jae Jo, Heon-Sang Jeong, Yun-Bae Kim

**Affiliations:** 1 College of Veterinary Medicine, Chungbuk National University, Cheongju 28644, Republic of Korea; 2 Gumi Smart Agriculture Research Institute, Gyeongsanbuk-do Agricultural Research & Extension Services, Gumi 39102, Republic of Korea; 3 High-Technology Research Institute, ThanEver Inc., Daejeon 34054, Republic of Korea; 4 College of Veterinary Medicine, Kangwon National University, Chuncheon 24341, Republic of Korea; 5 Department of Food Science and Biotechnology, Chungbuk National University, Cheongju 28644, Republic of Korea

**Keywords:** rosebud extract, antioxidant, pyrogallol (1,2,3-benzenetriol), antioxidative activity, anti-allergic activity, anti-inflammatory activity

## Abstract

Since chemical anti-allergic compounds have adverse effects, many investigators pay attention to relatively safe natural products. Twenty-four newly crossbred rosebuds were extracted with 80% ethanol and analyzed for polyphenols, flavonoids, tannins, proanthocyanidins, and pyrogallol (1,2,3-benzenetriol). The extracts’ antioxidative, anti-allergic, and anti-inflammatory activities were assessed in vitro and in vivo. Among candidates, Lover Shy, Pretty Velvet, Ice Wing, Red Perfume, Onnuri, Jaemina Red, and Hanggina were found to possess high concentrations of antioxidant components and antioxidative activity. By comparison, Pretty Velvet, Red Perfume, Jaemina Red, Hanggina, Onnuri, and Ice Wing were highly effective in anti-allergic and anti-inflammatory activities in vitro, in parallel with their concentrations of pyrogallol. Their anti-allergic effects were confirmed in mice: The six extracts protected against Compound 48/80-induced mortality and scratching behaviors in a dose-dependent manner. The allergen-induced increases in serum IgE and histamine, as well as inflammatory cytokines, tumor-necrosis factor-α, and interleukin-1β, were remarkably attenuated following treatment with the rosebud extracts. These findings suggest that the extracts and active ingredients from cross-bred rosebuds exert anti-allergic and anti-inflammatory activities through their high levels of pyrogallol and antioxidants, and that they could be promising candidates to overcome allergic responses such as atopic dermatitis.

## 1. Introduction

As an excessive immune function of the body, hypersensitivity reactions are classified into four types [1]. Among them, type 1 hypersensitivity reactions occur when immunoglobulin E (IgE), which is produced excessively through repeated exposure to allergens, binds to mast cells, and causes systemic anaphylactic shock or local itching and inflammation as granules containing histamines and chemotactic factors are released [2]. Representative type 1 hypersensitivity reactions include atopic dermatitis and respiratory asthma, which are mediated by Th2 cytokines. Atopic dermatitis is a chronic relapsing inflammatory skin disease, showing the highest prevalence in childhood. The number of atopic dermatitis patients in Korea was over 1 million in 2022 (Korea Health Insurance Review and Assessment Service) and has been increasing sharply every year. And the worldwide incidence of atopic dermatitis has increased by 2–3 times over the last 30 years [3].

As the cause of atopic dermatitis, genetic and environmental factors are involved. For example, atopic dermatitis is often caused by hypersensitivity reactions to dust, mites, bacteria, and fungi in the house, pollens, environmental chemicals, and chemicals added to food [4]. According to a review [5], allergic inflammations occur when external triggers, called allergens, penetrate impaired skin barriers and reach subcutaneous mast cells. Many inflammatory mediators, including histamine and chemotactic factors released from mast cells, cause itching symptoms. Itching symptoms lead to scratching behaviors and tissue damage, exacerbating skin inflammation so that an ‘itching–scratching’ vicious cycle is formed.

In clinical practice, antihistamines and steroids are commonly prescribed as therapeutic agents. Although antihistamines suppress itching symptoms caused by urticaria [6], they play only a limited role in the treatment of atopic dermatitis. Since long-term use of antihistamines can cause adverse effects, interest in natural products with high therapeutic effects and low side effects is increasing. The search for new functional substances from natural products has been accelerating after the enactment of the Korea Food and Drug Administration’s notification on safety and efficacy evaluation standards and approval procedures for functional foods and functional cosmetics [7].

Roses with various colors and shapes are a dicotyledonous plant belonging to the genus Rosa in the Rosaceae family. Rose flowers have been widely used for ornamental purposes, and rose oil has been widely used as a raw material for perfumes and basic cosmetics. By comparison with scent and oils, the nutritional and pharmacological activities of roses have not been studied enough. Roses were suggested as a therapeutic agent because they contain various phytochemicals, including antioxidants [8]. In fact, it was found that rose petals contain vitamins, polyphenols, anthocyanins, flavonoids, and lactones, and so they have a high antioxidative potential [9]. In addition, anthocyanins possessing anti-aging, cardioprotective, anti-cancer, and anti-diabetes activities were rich in rose extracts [10,11,12]. As additional advantages, roses are caffeine-free [13] and exhibit various anti-microbial activities [14].

Recently, we reported strong antioxidative and anti-inflammatory effects of rosebud extracts from new 24 rose cultivars [15]. In addition, we demonstrated that they exerted neuroprotective, anti-microbial, skin-whitening, wrinkle-improving, and anti-allergic properties [14,16,17]. Notably, it was found that pyrogallol is a major antioxidant that displays neuroprotective and anti-allergic effects, in which it suppressed interleukin-9 (IL-9) gene expression and calcineurin/nuclear factor of activated T-cells (NFAT) signaling [18,19,20,21,22]. In this context, we extended analyses of antioxidants and pyrogallol in 24 rosebud extracts, and assessed their anti-allergic and anti-inflammatory activities in vitro and in vivo, demonstrating protective potentials against systemic and local skin anaphylactic responses.

## 2. Materials and Methods

### 2.1. Preparation of Rosebud Extracts

Twenty-four newly crossbred roses were cultivated in an experimental nursery garden at the Gumi Smart Agriculture Research Institute (Gumi, Republic of Korea). Half-opened rosebuds were collected during May from 3-year-old trees raised at a controlled temperature (10–28 °C) in plastic greenhouses. The rose flowers were hot-air dried immediately after harvest at 70 °C for 16 h for full dryness.

**Figure 1:**
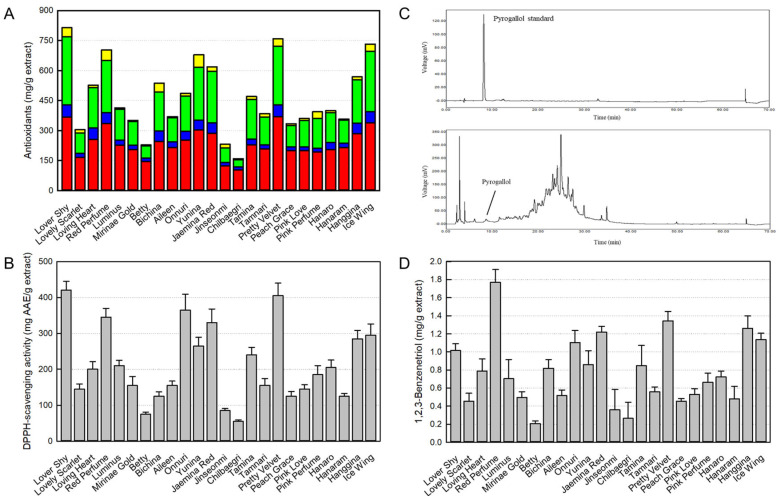
Antioxidant and pyrogallol (1,2,3-benzenetriol) content in rosebud extracts and their antioxidative activities. (**A**) Antioxidant content. Red: polyphenols, blue: flavonoids, green: tannins, and yellow: protoanthocyanidins. (**B**) DPPH-scavenging antioxidative activity. (**C**) HPLC chromatogram of pyrogallol standard and a rosebud extract. (**D**) Pyrogallol content in rosebud extracts.

Dried rosebuds from 24 new crossbred cultivars (*Rosa hybrida*) were used, i.e., (1) Lover Shy, (2) Lovely Scarlet, (3) Loving Heart, (4) Red Perfume, (5) Luminus, (6) Mirinae Gold, (7) Betty, (8) Bichina, (9) Aileen, (10) Onnuri, (11) Yunina, (12) Jaemina Red, (13) Jinseonmi, (14) Chilbaegri, (15) Tamina, (16) Tamnari, (17) Pretty Velvet, (18) Peach Grace, (19) Pink Love, (20) Pink Perfume, (21) Hanaro, (22) Hanaram, (23) Hanggina, and (24) Ice Wing (Figure 1).

Based on our previous studies, the rosebuds were extracted with 80% ethanol to achieve high antioxidant content [15]. The dried rosebuds were pulverized in a rotor mill (Laval Lab Inc., Laval, QC, Canada) and immersed in 80% ethanol in an ultrasonic water bath, in which the extraction solvent/solid ratio was set to 49:1 (980 mL, 80% ethanol/20 g dried rosebuds). The bath was heated at 60–70 °C for 2 h, and then ultrasonically extracted for 1 h. After extraction, the mixture was cooled at room temperature, filtered, and then concentrated to 60 brix under reduced pressure using a vacuum evaporator (Rotary Vacuum Evaporator N-N series; Eyela, Tokyo, Japan). The yields were found to be 20–28%. The samples were stored at 4 °C for a week until use.

### 2.2. Analysis of Antioxidative Components

#### 2.2.1. Analysis of Total Polyphenols

The total polyphenol content of the rosebud extracts was measured in triplicate according to the method of Dewanto et al. [15,23]. That is, the total polyphenol content was measured according to the principle that Folin–Ciocalteu’s phenol reagent is reduced by polyphenolic compounds in the extracts to develop a molybdenum color. The concentration of the samples was adjusted to 1 mg/mL, 2 mL of 2% Na_2_CO_3_ was added for reaction for 3 min, 100 µL of 50% Folin–Ciocalteu’s phenol reagent was added, the mixture was left alone for 30 min, and the absorbance of the reaction solution was measured at 750 nm. A standard calibration curve was prepared using gallic acid (Sigma-Aldrich, St. Louis, MO, USA) diluted to 10, 20, 30, 40, and 50 times the standard materials, and the polyphenol content was expressed as mg of gallic acid in 1 g of the sample.

#### 2.2.2. Analysis of Flavonoids

The total flavonoid content of the rosebud extracts was analyzed in triplicate using the method of Zhishen et al. [24] with some modifications. The concentration of the samples was adjusted to 1 mg/mL, 1 mL of distilled water, and 75 µL of 5% sodium nitrite were added to 250 µL of the sample solution, 150 µL of 10% aluminum chloride hydrate was added 5 min later, 500 µL of 1 M NaOH was added 6 min later, the mixture was left alone for 11 min, and the absorbance was measured at 510 nm. A standard calibration curve was prepared with (+)-catechin hydrate (Sigma-Aldrich), and the flavonoid content was expressed as mg of catechin in 1 g of the sample.

#### 2.2.3. Analysis of Tannins

The total tannin content of the rosebud extracts was measured in triplicate according to the method of Duval and Shetty [25]. The concentration of the sample was adjusted to 1 mg/mL, 1 mL of 95% ethanol and 1 mL of distilled water were added to 1 mL of the sample solution, the mixture was shaken well, 1 mL of 5% Na_2_CO_3_ solution and 0.5 mL of 1 N Folin–Ciocalteu’s reagent (Sigma-Aldrich) were added to the mixture, the color was developed at room temperature for 60 min, and the absorbance was measured at 725 nm. A standard calibration curve was prepared with tannic acid (Sigma-Aldrich), and the tannin content was expressed as mg of tannic acid in 1 g of the sample.

#### 2.2.4. Analysis of Proanthocyanidins

The total proanthocyanidin content of the rosebud extracts was measured in triplicate using the vanillin–sulfuric acid method modified according to the method of Takahama et al. [26]. The concentration of the sample was adjusted to 1 mg/mL, and 0.5 mL of 1.2% vanillin solution and 0.5 mL of 20% sulfuric acid were added to 0.2 mL of the sample solution; the mixture was left alone for 20 min, and the absorbance was measured at 500 nm using an ELISA reader (UV-1650PC; Shimadzu, Kyoto, Japan). A standard calibration curve was prepared with (+)-catechin (Sigma-Aldrich), and the proanthocyanidin content was expressed as mg of catechin in 1 g of the sample.

#### 2.2.5. Analysis of Pyrogallol (1,2,3-Benzenetriol)

The pyrogallol content of the rosebud extracts was analyzed in triplicate by HPLC (ACME 9000 system; Younglin, Anyang, Republic of Korea) [27]. Separation was performed on a YMC-Triart C18 column (4.6 × 250 mm; YMC, Kyoto, Japan). The mobile phase consisted of acetonitrile containing 0.1% acetic acid (A) and water (B), delivered under gradient elution conditions. The gradient conditions consisted of A:B as follows: initial 92:8 (%, *v*/*v*), 90:10 (from 2 min), 70:30 (from 27 min), 10:90 (from 50 min), 0:100 (from 51 min), 0:100 (from 60 min), and 92:9 (from 62 min). The flow rate was set at 1.0 mL/min with 20 °C of column temperature, and a 20 μL aliquot of each sample was injected. Detection was carried out using a UV detector at a wavelength of 280 nm. Pyrogallol (extra-pure grade) was obtained from Daejung Chemicals & Metals (Siheung, Republic of Korea). Quantification was performed by comparison with a calibration curve constructed using a pyrogallol standard.

### 2.3. Analysis of Antioxidative, Anti-Allergic, and Anti-Inflammatory Activities

#### 2.3.1. Measurement of DPPH-Scavenging Activity

Antioxidative efficacy of the rosebud extracts was measured in triplicate as described previously [15], based on the scavenging potential of 1,1-diphenyl-2-picrylhydrazyl (DPPH, Sigma-Aldrich). That is, 0.2 mL rose extract or distilled water (blank) was added to 0.8 mL DPPH solution (0.2 mM) to adjust to 0.1 mg/mL. After 30 min incubation, absorbance was measured at 520 nm. DPPH radical-scavenging activity, i.e., electron-donating ability, was expressed as mg AAE in 1 g extract with the difference in absorbance according to the addition and non-addition (distilled water) of the test substance.

#### 2.3.2. Measurement of Mast Cell Degranulation

The cell line RBL-2H3 (KCLB No. 22256, Lot No. 43077) was from the Korea Research Institute of Bioscience and Biotechnology (KRIBB) (Jeongeup, Republic of Korea). The cells were cultivated in a 5% CO_2_ incubator (37 °C), using a minimal essential medium (MEM) supplemented with 10% fetal bovine serum (FBS) and antibiotic–antimycotic (Gibco, Grand Island, NE, USA).

Mast cell degranulation was measured in triplicate with the release of β-hexosaminidase, which is a granule marker [16]. The cells were separated from the culture flask by treatment with an enzyme (trypsin-EDTA), and the cell suspension was centrifuged at 500× *g* for 5 min. Cell suspension was prepared at 2 × 10^6^ cells/mL, aliquoted (100 μL) into a 96-well cell culture plate, and stabilized for at least 3 h.

After discarding the culture medium, the wells were treated with the new culture medium containing anti-dinitrophenyl (DNP)-IgE (10 ng/mL) and cultivated overnight. The cells were treated with rosebud extracts (final concentrations of 10, 32, or 100 µg/mL) or standard pyrogallol compound (0.03–10 μg/mL), and incubated for 30 min. Thereafter, the cells were stimulated with DNP-human serum albumin (HSA; 10 µg/mL) to induce mast cell degranulation. After 1 h incubation, 60 µL of the supernatant was taken, mixed with 15 µL of substrate buffer, and made to react at 37 °C for 2 h. The stop solution (150 µL) was added, and the absorbance was measured at 405 nm.

#### 2.3.3. Measurement of Nitric Oxide (NO) Production

The inflammatory response of macrophages was measured in triplicate with NO production according to cell activation [15]. The cell line RAW 264.7 (ATCC TIB-71, KCLB No. 40071) used in the test was bought from KRIBB. The cells were cultivated overnight in a 96-well plate at a concentration of 1 × 10^7^ cells/mL. In order to activate cells, the cells were treated with lipopolysaccharide (LPS; 1 μg/mL). The cells were treated with rosebud extracts (10, 32, or 100 μg/mL) or standard pyrogallol compound (0.03–10 μg/mL), and incubated for 24 h. An equal volume of Griess reagent (Promega, Madison, WI, USA) was mixed, and the absorbance was measured at 540 nm 10 min later for NO measurement. A standard calibration curve was prepared with sodium nitrite, and the NO concentration in the culture medium was calculated.

### 2.4. Analysis of Correlation Between Ingredients and Activities

#### 2.4.1. Correlation Between Antioxidant Content and Antioxidative Activity

The correlations between antioxidant (polyphenol, flavonoid, tannin, or protoanthocyanidin) content and antioxidative activities were analyzed. Linear regression coefficients were calculated for the estimation of the relationship using a SigmaPlot program.

#### 2.4.2. Correlation Between Pyrogallol Content and Anti-Allergic Activity

The correlation between pyrogallol content in rosebud extracts or standard pyrogallol and anti-allergic activity was analyzed. The linear regression coefficient was calculated as described above.

#### 2.4.3. Correlation Between Pyrogallol Content and Anti-Inflammatory Activity

The correlation between pyrogallol content in rosebud extracts or standard pyrogallol and anti-inflammatory activity was analyzed. The linear regression coefficient was calculated as described above.

### 2.5. Measurement of Mouse Allergic Reaction

#### 2.5.1. Animals

Male ICR mice (6 weeks old, 25–28 g) for the induction of systemic and local hypersensitivity reactions were procured from Daehan Biolink (Eumseong, Republic of Korea) and housed in polysulfone cages (3 animals/cage). Only healthy animals were used after a 1-week acclimation to the laboratory environment. The environment of the animal laboratory was adjusted to a temperature of 23 ± 2 °C, relative humidity of 55 ± 10%, ventilation frequency of 12 times/hour, lighting cycle of 12 h (07:00~19:00), and illumination of 150~300 lux. They were fed standard rodent chow and purified water ad libitum. The animal experiments were conducted under the approval of the Institutional Animal Care and Use Committee (IACUC) of Chungbuk National University, and according to the Standard Operating Procedures (SOP) of the same institution.

#### 2.5.2. Measurement of Systemic Allergic Reaction (Death from Shock)

The anti-allergic activity of rosebud extracts on systemic anaphylactic shock was assessed in mice (*n* = 6/group under random grouping) challenged with Compound-48/80, a mast cell degranulator. Rosebud extracts were diluted in saline and intraperitoneally administered to mice at doses of 10, 30, or 100 mg/kg. Thirty min later, a lethal dose (8 mg/kg) of Compound 48/80 was intraperitoneally injected to induce an anaphylactic reaction, and thereafter the mortality rate of the mice was recorded for 60 min [16,28].

#### 2.5.3. Measurement of Local Allergic Reaction (Skin Itching)

In order to investigate the anti-allergic activity of rosebud extracts on atopic dermatitis, the extracts were diluted in purified water, orally administered to mice (n = 6/group under random grouping) at doses of 10, 30, or 100 mg/kg. Thirty min later, Compound 48/80 (50 μg/site, 50 μL at 1 mg/mL) was subcutaneously injected between the two shoulder blades [16,29,30]. Thereafter, the number of scratching behaviors was recorded for 60 min.

#### 2.5.4. Measurement of Blood IgE and Histamine

After deep anesthesia with diethyl ether, blood was collected from the abdominal vena cava into a Vacutainer (BD 367986; BD, Seoul, Republic of Korea) and centrifuged at 3000 rpm for 15 min to obtain serum. Serum IgE and histamine were determined via ELISA according to the manufacturer’s instructions. Briefly, serum was loaded into ELISA wells together with antibodies specific for IgE (K3231081; Komabiotech, Seoul, Republic of Korea) or histamine (ab213975; Abcam, Cambridge, UK), and incubated for 0.5–1 h at room temperature. After washing, the secondary antibody was treated and incubated for 30 min. Following treatment with a substrate for color development, absorbance was measured at 450 nm [30].

#### 2.5.5. Measurement of Blood Cytokines

As inflammatory cytokines, the levels of serum TNF-α and IL-6 were measured using mouse Quantikine immunoassay kits (R&D Systems, Minneapolis, MN, USA) according to the ELISA procedures provided by the manufacturer (Molecular Devices, San Jose, CA, USA) [30,31].

### 2.6. Statistical Analysis

Data are presented as mean ± standard deviation. Statistical analysis was performed with SPSS version 26.0 program (SPSS Inc., Chicago, IL, USA). Differences among groups were analyzed with one-way ANOVA, followed by Tukey’s HSD at a level of *p* < 0.05. Correlations between ingredients and their activities were analyzed with SigmaPlot Version 12.5 (Grafiti, Palo Alto, CA, USA).

## 3. Results

### 3.1. Antioxidant Contents in Rosebud Extracts and Their Antioxidative Activities

We extensively analyzed antioxidants, including polyphenols, flavonoids, tannins, and proanthocyanidins, from 24 rose cultivars. Among these four ingredients, polyphenols were very rich in Pretty Velvet (368.5 ± 1.5 mg/g), Lover Shy (367.5 ± 5.6 mg/g), Ice Wing (338.4 ± 2.2 mg/g), Red Perfume (335.2 ± 2.1 mg/g), Yunina (302.4 ± 4.7 mg/g), and Jaemina Red (286.5 ± 2.1 mg/g) (Figure 1A). Flavonoids were lower than polyphenols, but Lover Shy (61.3 ± 2.0 mg/g), Pretty Velvet (60.0 ± 5.1), Loving Heart (57.0 ± 3.3 mg/g), Ice Wing (55.3 ± 1.4 mg/g), Red Perfume (54.0 ± 0.8 mg/g), Bichina (52.3 ± 0.3 mg/g), and Haggina (52.0 ± 1.3 mg/g) were found to contain relatively high concentrations. Tannins were high in Lover Shy (339.6 ± 1.3 mg/g), Ice Wing (302.3 ± 3.8 mg/g), Pretty Velvet (293.6 ± 4.2 mg/g), Yunina (263.7 ± 0.2 mg/g), Red Perfume (261. 3± 1.3 mg/g), and Jaemina Red (258.5 ± 1.4 mg/g). Protoanthocyanidins were relatively low among other components, but Yunina (62.1 ± 0.8 mg/g), Red Perfume (52.0 ± 2.5 mg/g), Lover Shy (45.6 ± 3.5 mg/g), Bichina (43.8 ± 1.7 mg/g), Ice Wing (36.7 ± 1.4 mg/g), and Pretty Velvet (36.5 ± 2.1 mg/g) had high protoanthocyanidin concentrations.

There were big differences in total antioxidant contents, ranging from 814.0 mg/g (Lover Shy) to 159.9 mg/g (Chilbaegri). Among 24 rosebud extracts, Lover Shy (814.0 mg/g), Pretty Velvet (758.6 mg/g), Ice Wing (723.1 mg/g), Red Perfume (698.2 mg/g), Onnuri (678.5 mg/g), Jaemina Red (618.4 mg/g), and Hanggina (568.3 mg/g) were found to contain high concentrations of the antioxidants.

Notably, the overall antioxidative activities of 24 rosebud extracts showed a similar trend for their antioxidative content (Figure 1B). The correlation between antioxidants and their antioxidative activities is described below.

### 3.2. Identification and Content of Pyrogallol in Rosebud Extracts

Next, we analyzed pyrogallol, as an anti-allergic ingredient, in 24 rosebud extracts. As a result of HPLC analysis, pyrogallol standard was detected at a retention time of 8.5450 min, and pyrogallol was detected at the same time in a rosebud extract (Figure 1C).

There was a difference in pyrogallol concentration ranging from 1.77 ± 0.14 mg/g (Red Perfume) to 0.21 ± 0.03 mg/g (Betty) (Figure 1D). Among 24 rose cultivars, the concentration of pyrogallol was highest in Red Perfume (1.77 ± 0.14 mg/g), Pretty Velvet (1.34 ± 0.10 mg/g), Hanggina (1.26 ± 0.14 mg/g), Jaemina Red (1.22 ± 0.06 mg/g), Ice Wing (1.14 ± 0.07 mg/g), Onnuri (1.10 ± 0.13 mg/g), and Lover Shy (1.02 ± 0.07 mg/g). Notably, the order of pyrogallol concentration was similar to the antioxidant content, although Lover Shy showed relatively low pyrogallol levels compared with the highest antioxidants.

### 3.3. Inhibition of Mast Cell Degranulation

**Figure 2:**
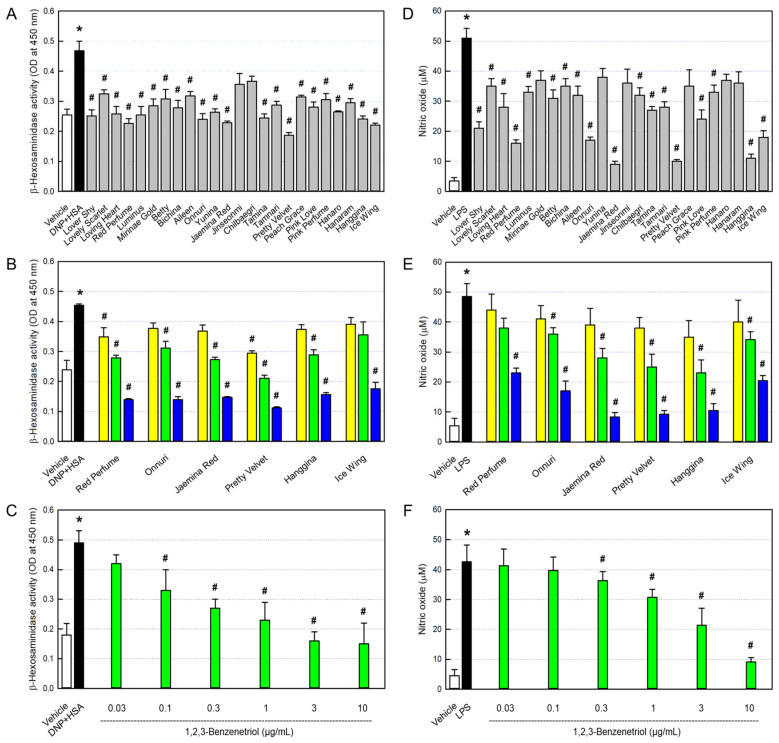
Anti-allergic (**A**–**C**) and anti-inflammatory (**D**–**F**) activities of rosebud extracts and standard pyrogallol compound (1,2,3-benzenetril). (**A**) Inhibition by 24 rosebud extracts of β-hexosaminidase release from RBL-2H3 mast cells at 100 μg/mL. (**B**) Concentration-dependent inhibition of β-hexosaminidase release by selected rosebud extracts at concentrations of 10 (yellow), 32 (green), or 100 (blue) μg/mL. (**C**) Concentration-dependent inhibition of nitric oxide production by standard pyrogallol compound. (**D**) Inhibition by 24 rosebud extracts of nitric oxide production from RAW 264.7 macrophages at 100 μg/mL. (**E**) Concentration-dependent inhibition of nitric oxide production by selected rosebud extracts at concentrations of 10 (yellow), 32 (green), or 100 (blue) μg/mL. (**F**) Concentration-dependent inhibition of β-hexosaminidase release by standard pyrogallol compound. * Significantly different from Vehicle control (*p* < 0.05). ^#^ Significantly different from DNP-HSA or LPS alone (*p* < 0.05).

In order to assess allergic reaction, we analyzed β-hexosaminidase, instead of histamine, released from mast cells sensitized with IgE, since both of them are secreted by allergic reactions such as asthma, rhinitis, and atopic dermatitis [16]. β-Hexosaminidase release from RBL-2H3 cells markedly increased following sensitization with anti-DNP IgE (Figure 2A). However, such an IgE-mediated increase in β-hexosaminidase release was attenuated by rosebud extracts (100 μg/mL), in which Red Perfume, Onnuri, Jaemina Red, Pretty Velvet, Hanggina, and Ice Wing were the most effective, reducing it to a normal level. Notably, the anti-allergic activity was in parallel with the concentration of pyrogallol in the rosebud extracts (Figure 1D). Furthermore, we assessed the concentration-activity at 10, 30, and 100 μg/mL. As a result, the six selected extracts, Red Perfume, Onnuri, Jaemina Red, Pretty Velvet, Hanggina, and Ice Wing, displayed concentration-dependent anti-allergic activities (Figure 2B). Notably, β-hexosaminidase release was inhibited by the standard pyrogallol compound in a concentration-dependent manner at concentrations of 0.1–10 μg/mL (Figure 2C) without cytotoxicity (Supplementary Figure S1).

### 3.4. Inhibition of Macrophage NO Production

As an inflammatory parameter, we analyzed NO production from macrophages activated with LPS. Treatment of RAW 264.7 cells with LPS greatly increased NO release (Figure 2D). However, all 24 rosebud extracts inhibited the NO production, wherein Red Perfume, Onnuri, Jaemina Red, Pretty Velvet, Hanggina, and Ice Wing were highly effective. Interestingly, the anti-inflammatory activity was in parallel with the concentrations of antioxidants and pyrogallol in the rosebud extracts (Figure 1A,D). Furthermore, the six selected extracts, Red Perfume, Onnuri, Jaemina Red, Pretty Velvet, Hanggina, and Ice Wing, exhibited concentration-dependent anti-inflammatory activities at 10, 30, and 100 μg/mL (Figure 2E). The NO production was also inhibited by the standard pyrogallol compound at concentrations of 0.3–10 μg/mL (Figure 2F) without cytotoxicity (Figure S2).

### 3.5. Analysis of Correlation Between Ingredients and Antioxidative Activity

**Figure 3:**
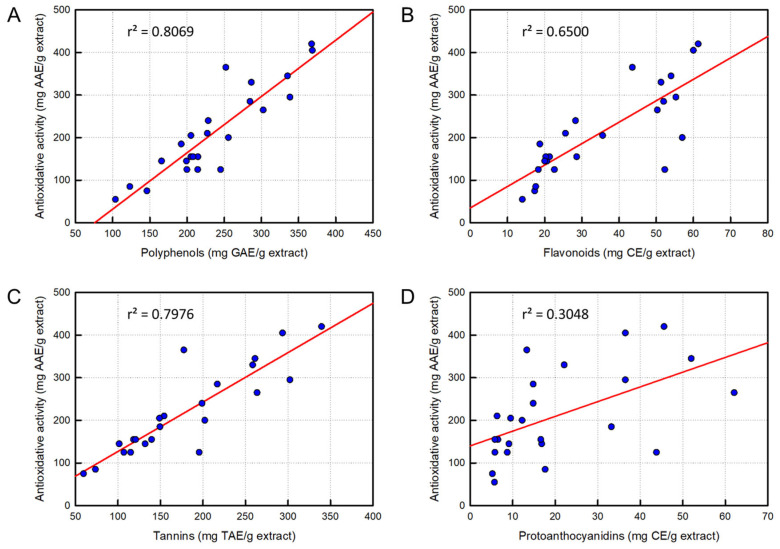
Correlations between 4 antioxidants in rosebud extracts and antioxidative activity at 100 μg/mL. (**A**) Linear regression of correlation between polyphenol content and DPPH-scavenging activity. (**B**) Linear regression of correlation between flavonoid content and DPPH-scavenging activity. (**C**) Linear regression of correlation between tannin content and DPPH-scavenging activity. (**D**) Linear regression of correlation between protoanthocyanidin content and DPPH-scavenging activity.

We analyzed correlations between antioxidant contents and DPPH radical-scavenging activities to understand which ingredients are closely related to the antioxidative effect (Figure 3). As a result of linear regression analysis, polyphenol content displayed a good correlation (r^2^ = 0.8069). In addition, flavonoids (r^2^ = 0.6500) and tannins (r^2^ = 0.7976) exhibited a high relationship, but proanthocyanidins showed a relatively low correlation (r^2^ = 0.3048).

### 3.6. Analysis of Correlation Between Pyrogallol and Anti-Allergic or Anti-Inflammatory Activity

**Figure 4:**
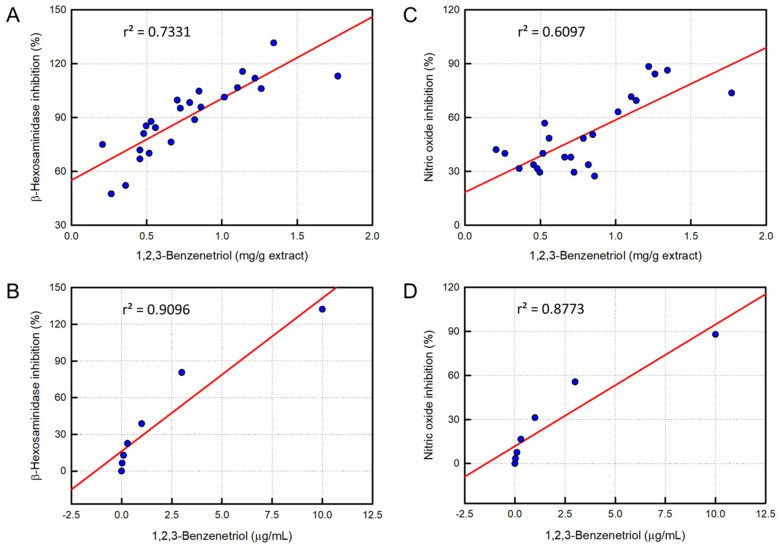
Correlations between pyrogallol (1,2,3-benzenetriol) in rosebud extracts (**A**,**B**) or standard pyrogallol compound (**C**,**D**) and anti-allergic (**A**,**C**) or anti-inflammatory (**B**,**D**) activity. (**A**) Linear regression of correlation between pyrogallol content in rosebud extracts and β-hexosaminidase release-inhibiting activity at 100 μg/mL. (**B**) Linear regression of correlation between pyrogallol content in rosebud extracts and nitric oxide production-inhibiting activity at 100 μg/mL. (**C**) Linear regression of correlation between standard pyrogallol compound and β-hexosaminidase release-inhibiting activity. (**D**) Linear regression of correlation between standard pyrogallol compound and nitric oxide production-inhibiting activity.

In the linear regression analysis between pyrogallol content in the rosebud extracts and anti-allergic activity, a good correlation (r^2^ = 0.7331) was attained (Figure 4A). NO production-inhibitory activity was also closely related to the pyrogallol content in the extracts (r^2^ = 0.6097) (Figure 4B).

In addition, the standard pyrogallol exhibited a much higher correlation (r^2^ = 0.9096) with anti-allergic activity (Figure 4C). Furthermore, the pyrogallol compound was found to have a high correlation (r^2^ = 0.8773) with anti-inflammatory activity (Figure 4D).

### 3.7. Inhibition of Systemic and Skin Allergic Reactions

**Figure 5:**
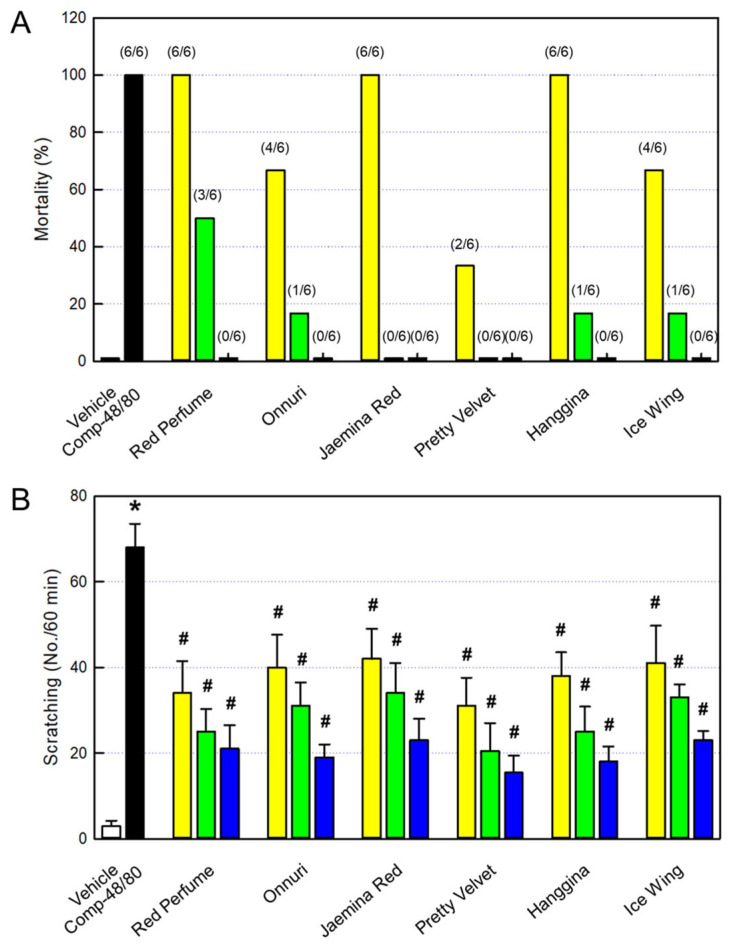
Anti-allergic activities of rosebud extracts in mice. (**A**) Protection by rosebud extracts against systemic anaphylactic shock (mortality) of Compound-48/80-challenged mice (died/challenged). (**B**) Inhibition by rosebud extracts of skin allergic reaction (scratching behaviors) in Compound-48/80-challenged mice. Yellow: 10 mg/kg, green: 30 mg/kg, and blue: 100 mg/kg. * Significantly different from Vehicle control (*p* < 0.05). # Significantly different from Compound-48/80 alone (*p* < 0.05).

All the mice (n = 6/group) intraperitoneally challenged with a lethal dose (8 mg/kg) of Compound-48/80 died within 60 min due to a systemic shock reaction (Figure 5A). Notably, 30 min intraperitoneal pretreatment with six rosebud extracts (10, 30, or 100 mg/kg) protected against the Compound-48/80-induced mortality in a dose-dependent manner. In a relative potency, Pretty Velvet, Onnuri, and Ice Wing were somewhat more effective than Jaemina Red, Hanggina, and Red Perfume, although all the mice survived at the high dose (100 mg/kg) of each rosebud extract.

Based on the systemic anti-allergic activity of rosebud extracts, we tried to confirm their effects on skin allergic reactions. The mice subcutaneously injected with Compound-48/80 (50 μg/site) showed severe itching symptoms, scratching 68.0 ± 5.5 times for 60 min (Figure 5B). However, 30 min oral pretreatment with the six rosebud extracts, Red Perfume, Onnuri, Jaemina Red, Pretty Velvet, Hanggina, and Ice Wing, significantly attenuated the scratching behavior in a dose-dependent manner.

### 3.8. Inhibition of Blood Allergic and Inflammatory Parameters

**Figure 6:**
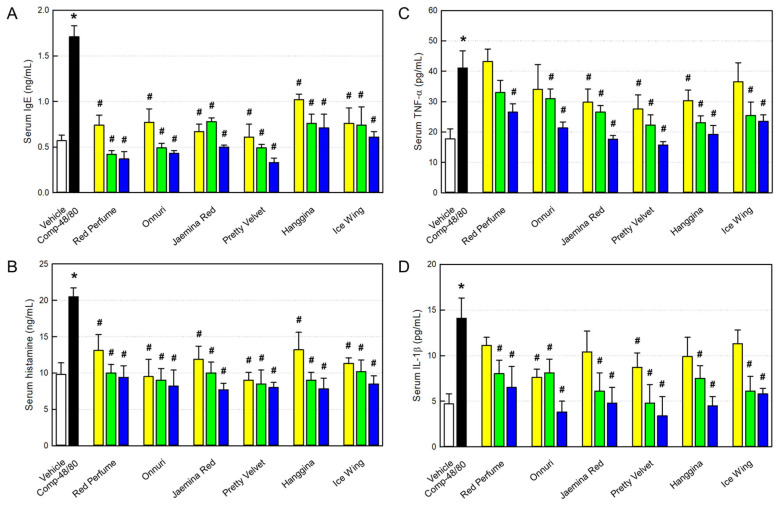
Inhibition by rosebud extracts of blood allergic (**A**,**B**) and inflammatory (**C**,**D**) parameters in Compoud-48/80-challenged mice. (**A**) IgE. (**B**) Histamine. (**C**) Tumor-necrosis factor-α (TNF-α). (**D**) Interleukin-1β (IL-1β). Yellow: 10 mg/kg, green: 30 mg/kg, and blue: 100 mg/kg. * Significantly different from Vehicle control (*p* < 0.05). # Significantly different from Compound-48/80 alone (*p* < 0.05).

According to the anti-allergic activity of the rosebud extracts, we analyzed blood IgE and histamine from the mice subcutaneously challenged with Compound-48/80 to clarify underlying mechanisms. The serum IgE level of the mice markedly increased after challenge with Compound-48/80 (Figure 6A). As a related mediator, serum histamine also significantly increased as an allergic response (Figure 6B). It is interesting to note that the six rosebud extracts inhibited both the IgE and histamine levels, especially to lower blood concentrations than normal levels at high doses.

As markers of inflammation, we analyzed blood TNF-α and IL-1β, the major inflammatory cytokines. As expected, both the cytokines were greatly enhanced by challenge with Compound-48/80 (Figure 6C,D). However, the six rosebud extracts were found to lower both the blood TNF-α and IL-1β levels to normal levels at high doses, as inferred from the inhibitory effects on the NO production from RAW 264.7 macrophages (Figure 2D,E).

## 4. Discussion

Most animals consuming oxygen in metabolism inevitably suffer from tissue injury and aging due to the generation of reactive oxygen species (ROS), called oxidative stress [32]. In order to counteract ROS toxicity, living organisms synthesize or ingest antioxidant molecules. When oxidative stress exceeds the antioxidant defense system, theoretically, the oxidative damage can be attenuated by ingesting antioxidants.

Antioxidative compounds present in plants, that is, phytochemicals, are largely divided into phenolics, carotenoids, alkaloids, and organosulfur- or nitrogen-containing compounds. Among them, polyphenols, that is, phenolics, account for the largest part. In particular, among phenolics, flavonoids that have strong antioxidant activity are well known to prevent and delay aging by playing a role in preventing oxidative stress through the inhibition of lipid peroxidation [3]. Flavonoids are mainly composed of anthocyanidins, flavonols, flavones, catechins, and flavanones, and it has been demonstrated that most of the flavonoids have strong antioxidative and anti-bacterial activities depending on their structures [33]. Tannins are a sort of polyphenol that have an astringent taste. Originally, tannin was a word referring to substances used as preservatives when skins of animals are made into leather, but it is now a general term for large polyphenolic compounds that have sufficient hydroxyl groups and bind strongly to proteins or other macromolecules [34]. Proanthocyanidins and anthocyanins, well known as red pigments in plants, reduce oxidative stress, aging, and heart dysfunction with their free radical-scavenging abilities [35].

In our previous study, Colorado rose petal extract was found to contain high concentrations of polyphenols and flavonoids, and exhibited potential antioxidative and anti-inflammatory activities [18,22]. As expected, the extract protected against epileptic brain damage of mice challenged with excitotoxic kainic acid. Thereafter, we prepared rosebud extracts from 24 newly crossbred roses, and they showed good correlations between polyphenol content and antioxidative potency as well as between polyphenol concentration and anti-inflammatory activity in vitro [15]. Specifically, an extract from Pretty Velvet rose exhibited strong in vivo anti-inflammatory effects in a subcutaneous air-pouch inflammation model through steroid- and non-steroidal anti-inflammatory drug (NSAID)-like activities.

In the present study, we extended analyses to polyphenols, flavonoids, tannins, and proanthocyanidins, and demonstrated their anti-inflammatory effects, showing a good relationship with the antioxidant contents. Indeed, there were good correlations between antioxidant contents and their antioxidative activities in the order of polyphenols (r^2^ = 0.8069) > tannins (r^2^ = 0.7976) > flavonoids (r^2^ = 0.6500) >> proanthocyanidins (r^2^ = 0.3048). Notably, the content of polyphenols was the highest, followed by tannins, flavonoids, and protoanthocyanidins, although there were some differences in rose cultivars. Therefore, polyphenols and tannins are believed to mainly contribute to antioxidative activity.

Tissue damage caused by ROS is involved in most inflammatory reactions and also promotes immune hypersensitivity reactions, that is, systemic and local allergic reactions. Nowadays, opportunities to be exposed to various allergens are rapidly increasing, such as, for example, fine dust from reckless development and combustion of petrochemicals, house dust mites due to carpet culture, animal hair due to the increase in companion animal population, spring pollen, and new foreign fruits and foods [1,36,37,38,39]. Among them, asthma and atopic dermatitis, which are type 1 hypersensitivity reactions, are emerging as social problems. Mast cells are distributed in most organs and tissues, and they are important triggering cells for immune reactions such as allergy and anaphylaxis. In particular, an anaphylaxis reaction is induced by histamine and chemotactic factors released from mast cells as well as various inflammatory cytokines from other inflammatory cells [38,40,41].

Steroids are widely prescribed to treat atopic dermatitis, which is emerging as a major disease of modern people. But they should be used in an appropriate dose and in good balance because of their wide adverse effects, such as osteoporosis, osteonecrosis, myopathy, peptic ulcer, arteriosclerosis, dyslipidemia, acne, and skin atrophy [42]. In addition, the US Food and Drug Administration (FDA) required carcinogenicity warnings to be attached to Elidel^®^ and Protopic^®^, which are non-steroidal atopy treatments, and the Ministry of Food and Drug Safety (MFDS) of Korea restricts their use in young patients. Therefore, social demand for the development of safe therapeutic agents is rapidly increasing, and natural products may fulfill this requirement.

Earlier, we demonstrated that a rose petal extract has a strong anti-allergic property, inhibiting β-hexosaminidase release from RBL-2H3 cells as well as systemic and cutaneous anaphylaxis reactions in animals [16]. Notably, we found out that pyrogallol is a major antioxidant that displays neuroprotection in an animal model of ischemic stroke [18,19]. Actually, pyrogallol was found to have anti-allergic activity, alleviating nasal symptoms, including sneezing, in toluene-2,4-diisocyanate (TDI)-sensitized rats by suppressing Th2 cytokine expression calcineurin/nuclear factor of activated T-cell (NFAT) signaling [20,21].

In the present study, therefore, we analyzed pyrogallol content in 24 rosebud extracts and assessed their β-hexosaminidase- and NO-blocking activities. Indeed, there were good correlations between pyrogallol content and their anti-allergic (r^2^ = 0.7331) and anti-inflammatory (r^2^ = 0.6097) potentials. Therefore, it was confirmed that pyrogallol significantly contributes to anti-allergic and anti-inflammatory activities.

Next, we selected six extracts (Red Perfume, Onnuri, Jaemina Red, Pretty Velvet, Hanggina, and Ice Wing) showing strong anti-allergic and anti-inflammatory activities in vitro, and investigated their effects on systemic and local (skin) anaphylactic reactions in vivo. As inferred from the blocking activity on β-hexosaminidase release, the six rosebud extracts protected against Compound-48/80 lethality in a dose-dependent manner. Furthermore, the extracts markedly attenuated skin-scratching behaviors in mice challenged with Compound-48/80, as shown in a previous study [16]. Such anti-allergic effects of rosebud extracts were supported by blood IgE, histamine, and inflammatory cytokines. Indeed, serum IgE, histamine, TNF-α, and IL-1β were reduced following the rosebud extracts in parallel with the dose-dependent patterns in itching symptoms.

Roses have been widely used for decoration or as a component of perfumes because of their beautiful appearance and strong scent. In particular, Turkey, Bulgaria, Morocco, and China produce rose oil in large quantities and supply it as a raw material for cosmetics all over the world, and more recently, petals are used in jams or desserts [43]. However, the pharmacological activities of rose oil are not well-demonstrated. Rather, we have reported various effects such as antioxidation, anti-allergy, skin whitening, wrinkle improvement, anti-microbial, anti-inflammation, and neuroprotection of non-oil solvent extracts [14,15,16,17,18,19,44,45]. In the present study, we extended insights into the major component (pyrogallol) as an anti-allergic molecule of 24 new cultivars manufactured at the Korea Floriculture Research Institute. Based on the research results, it is expected that we can develop more effective medicinal plants possessing higher pyrogallol content for the management of allergic diseases such as atopic dermatitis.

## 5. Conclusions and Limitations

We demonstrated that 24 rosebud extracts displayed anti-allergic, anti-inflammatory, and antioxidative effects that are potentially associated with their ingredients, including pyrogallol and/or antioxidant phytochemicals. Specifically, pyrogallol was found to play a meaningful role in anti-anaphylactic activity. Therefore, it is proposed that the extracts and active ingredients from cross-bred rosebuds exert beneficial roles through their high levels of pyrogallol and antioxidants, and that those could be possible candidates to overcome allergic responses.

However, there are clear limitations in the present study. Too many phytochemical ingredients may be cross-involved in antioxidative, anti-allergic, and anti-inflammatory activities. Deeper molecular mechanisms should be elucidated for direct functional activities. In addition, it is necessary to find out the alterations of ingredients and their activities influenced by the cultivation environment, extraction methods that include solvents, and storage conditions in future studies. In spite of the broad limitations, the findings in this study show the genetic differences in functional compounds in rose cultivars, leading us to expect further development of anti-allergic and anti-inflammatory medicinal plants.

## Data Availability

The original contributions presented in the study are included in the article; further inquiries should be directed to the corresponding author.

## Supplementary Materials

The following supporting information can be downloaded at: https://www.mdpi.com/article/10.3390/cimb48050448/s1.

## Abbreviations

The following abbreviations are used in this manuscript:

AAE	Ascorbic acid equivalent
DNP	Anti-dinitrophenyl
DPPH	1,1-diphenyl-2-picrylhydrazyl
ELISA	Enzyme-linked immunosorbent assay
FBS	Fetal bovine serum
FDA	Food and Drug Administration
HSA	Human serum albumin
IACUC	Institutional Animal Care and Use Committee
IgE	Immunoglobulin E
IL-1β	Interleukin-1β
IL-9	Interleukin-9
LPS	Lipopolysaccharide
MEM	Minimal essential medium
MFDS	Ministry of Food and Drug Safety
NFAT	Nuclear factor of activated T-cells
NO	Nitric oxide
NSAID	Non-steroidal anti-inflammatory drugs
ROS	Reactive oxygen species
SOP	Standard operating procedures
TDI	Toluene-2,4-diisocyanate
TNF-α	Tumor-necrosis factor-α
