# Supplementary material for: Pyrogallol Contributes to the Anti-Allergic and Anti-Inflammatory Activities of Rosebud Extracts of Newly Crossbred Roses
Source: Curr Issues Mol Biol. 2026 Apr 25;48(5):448. doi: 10.3390/cimb48050448 (PMC13204907; doi:10.3390/cimb48050448)
Supplement: Supplementary file 1 [file cimb-48-00448-s001.zip › cimb-4194462-supplementary.pdf]

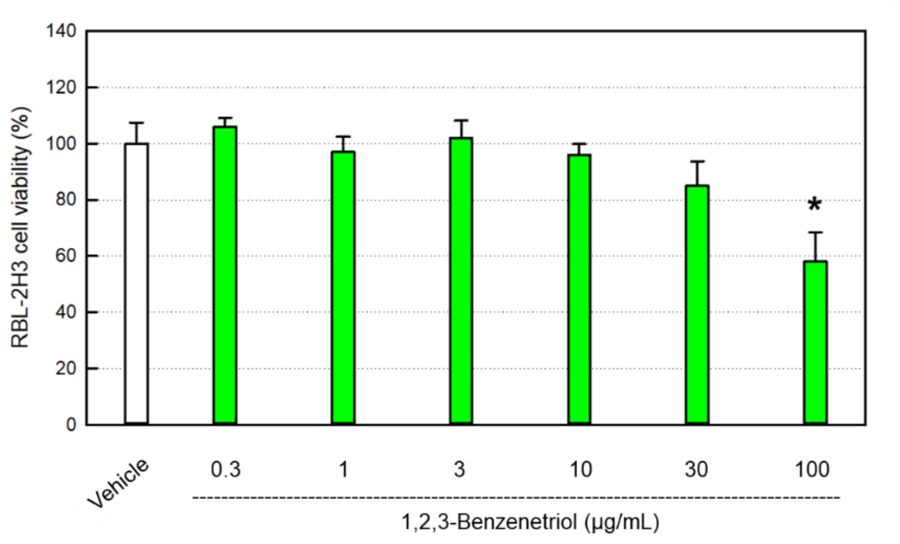

Figure S1. Cytotoxicity of pyrogallol (1,2,3-benzenetriol) in RBL-2H3 cells.

*Significantly different from Vehicle control ($P < 0.05$).

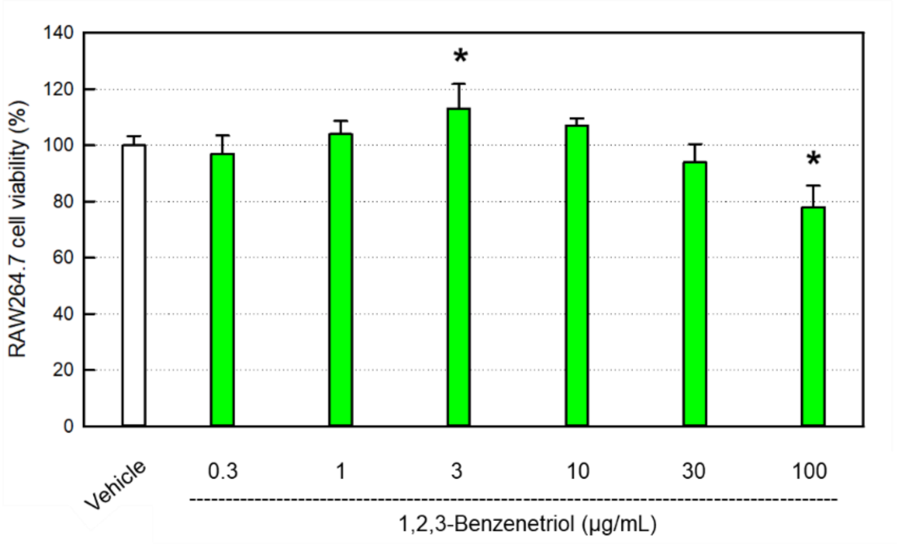

Figure S2. Cytotoxicity of pyrogallol (1,2,3-benzenetriol) in RAW264.7 cells.

*Significantly different from Vehicle control ($P < 0.05$).
